# Stem Cells as a Model of Study of SARS-CoV-2 and COVID-19: A Systematic Review of the Literature

**DOI:** 10.1155/2021/9915927

**Published:** 2021-08-25

**Authors:** María Verónica Cuevas-Gonzalez, Álvaro Garcia-Perez, Álvaro Edgar Gonzalez-Aragon Pineda, León Francisco Espinosa-Cristobal, Alejandro Donohue-Cornejo, Karla Lizette Tovar-Carrillo, Rosa Alicia Saucedo-Acuña, Juan Carlos Cuevas-Gonzalez

**Affiliations:** ^1^Research Division, National Autonomous University of Mexico, Mexico City, Mexico; ^2^Faculty of Higher Studies (FES) Iztacala, National Autonomous University of Mexico, Mexico City, Mexico; ^3^Institute of Biomedical Sciences, Autonomous University of Ciudad Juarez, Juarez City, Chihuahua, Mexico

## Abstract

**Background:**

The SARS-CoV-2 virus is the cause of the latest pandemic of the 21st century; it is responsible for the development of COVID-19. Within the multiple study models for both the biology and the treatment of SARS-CoV-2, the use of stem cells has been proposed because of their ability to increase the immune response and to repair tissue. Therefore, the objective of this review is to evaluate the role of stem cells against SARS-CoV-2 and COVID-19 in order to identify their potential as a study model and as a possible therapeutic source against tissue damage caused by this virus. Therefore, the following research question was established: What is the role of stem cells in the study of SARS-CoV-2 and the treatment of COVID-19?

**Materials and Methods:**

A search was carried out in the electronic databases of PUBMED, Scopus, and ScienceDirect. The following keywords were used: “SARS-CoV-2,” “COVID-19,” and “STEM CELL,” plus independent search strategies with the Boolean operators “OR” and “AND.” The identified reports were those whose main objective was the study of stem cells in relation to SARS-CoV-2 or COVID-19. For the development of this study, the following inclusion criteria were taken into account: studies whose main objective was the study of stem cells in relation to SARS-CoV-2 or COVID-19 and clinical case studies, case reports, clinical trials, pilot studies, in vitro, or in vivo studies. For assessment of the risk of bias for in vitro studies, the SciRAP tool was used. The data collected for each type of study, clinical or in vitro, were analyzed with descriptive statistics using the SPSS V.22 program.

**Results:**

Of the total of studies included (*n* = 39), 22 corresponded to in vitro investigations and 17 to human studies (clinical cases (*n* = 9), case series (*n* = 2), pilot clinical trials (*n* = 5), clinical trials (*n* = 1)). In vitro studies that induced pluripotent stem cells were the most used (*n* = 12), and in clinical studies, the umbilical stem cells derived were the most reported (*n* = 11). The mean age of the study subjects was 58.3 years. After the application of stem cell therapy, the follow-up period was 8 days minimum and 90 days maximum. *Discussion*. The mechanism by which the virus enters the cell is through protein “S,” located on the surface of the membrane, by recognizing the ACE2 receptor located on the target cell. The evidence that the expression of ACE2 and TMPRSS2 in stem cells indicates that stem cells from bone marrow and amniotic fluid have very little expression. This shows that stem cell has a low risk of infection with SARS-CoV-2.

**Conclusion:**

The use of stem cells is a highly relevant therapeutic option. It has been shown in both in vitro studies and clinical trials that it counteracts the excessive secretion of cytokines. There are even more studies that focus on long-term follow-up; thus, the potential for major side effects can be analyzed more clearly. Finally, the ethical use of stem cells from fetal or infant origin needs to be regulated. The study was registered in PROSPERO (no. CRD42021229038). The limitations of the study were because of the methodology employed, the sample was not very large, and the follow-up period of the clinical studies was relatively short.

## 1. Introduction

The SARS-CoV-2 virus was described for the first time in December 2019. It is the cause of the latest pandemic of the 21st century, and its spread throughout the world has been rapid, generating millions of infections and deaths. The affectation of this virus is responsible for the development of COVID-19, which is characterized from causing mild damage involving fever, cough, and dyspnea, to severe pneumonia, myocardial damage, arrhythmias, neurological damage, and multiple organ failure [1]. One of the main organs damaged is the liver, which, after infection with SARS-CoV-2, increases the levels of alanine aminotransferase, aspartate aminotransferase, lactic dehydrogenase, gamma glutamyl transferase, and total bilirubin given the cirrhotic poor immune function [2]. On the other hand, the microbiota plays a very relevant role in the severity of COVID-19 because coinfections by *Candida albicans* and *Candida glabrata* have been reported, contributing to the affectation of the immune system [3]. Due to these tragic effects, multiple research centers around the world have focused on understanding the biology of the virus as well as the treatment of the various side effects generated by SARS-CoV-2.

Stem cells are found within multiple study models for both the biology and treatment of SARS-CoV-2. These types of cells are unspecialized; they have the great advantage of being able to give rise to cells from specialized tissues and have the ability for self-renewal. According to their origin, these types of cells are found in fetuses, infants, and adults [4]. Another main characteristic is their ability to increase the immune response and repair tissue [5, 6], for which they have become a great source of relevant study of for the understanding and treatment of COVID-19. Therefore, the main objective of this work is to evaluate the role of stem cells against SARS-CoV-2 and COVID-19 in order to identify their potential as a study model and a possible therapeutic source against tissue damage caused by SARS-CoV-2.

To carry out this work, original studies were considered; clinical case reports, case series, and clinical trials were included, as well as basic science studies in vitro, in vivo, or in silico that covered the use of stem cells in relation to the SARS-CoV-2.

## 2. Materials and Methods

### 2.1. Research Question

What is the role of stem cells in the study of SARS-CoV-2 and the treatment of COVID-19?

### 2.2. Criteria Selection

#### 2.2.1. Inclusion Criteria

1. Studies whose main objective was the study of stem cells in relation to SARS-CoV-2 or COVID-19
2. Clinical case studies, case reports, clinical trials, pilot studies, in vitro, or in vivo studies

#### 2.2.2. Exclusion Criteria

- Literature reviews, systematic reviews, or meta-analysis, as well as studies in a language other than English or Spanish

Studies were separated according to their focus: in vitro or clinical studies. All the studies that met the established criteria were placed in a bibliographic manager (Mendely) for further analysis.

### 2.3. Search Strategy

A search was carried out in the electronic databases of PUBMED, Scopus, and ScienceDirect, and the following keywords were used: “SARS-CoV-2,” “COVID-19,” and “STEM CELL” employee independent search strategies with the Boolean operators “OR” and “AND.” Due to the importance of the topic, no time limit was established for the search; the last was March 2020.

A further search of the references of the selected studies was performed to detect potential studies that met the selection criteria.

### 2.4. Study Selection

For study selection, an initial filter was performed by title and abstract, which mentioned the study of stem cells related to SARS-CoV-2 and/or COVID-19. The selected studies were placed in a database in which a second full-text filter was performed identifying a specific bibliography that met the established selection criteria. The selection of the studies was carried out independently by two examiners (Cuevas-Gonzalez JC and Cuevas Gonzalez MV); in case of discrepancy, a third evaluator participated (Donohue-Cornejo A).

### 2.5. Data Extraction and Analysis

The data recollected were specific for each type of study; clinical or in vitro, the information gathered from in vitro studies was author, country, study type, stem cell origin, objective of the study, and conclusion. Additionally, in clinical studies, the data were number of subjects, mean age (years), treatment plan, and follow-up time after treatment administration. The data were placed in a database, and statistics were made based measures of central tendency. Descriptive statistics of the data were performed using the SPSS V.22 program.

### 2.6. Risk of Bias

To determine the risk of bias, different tools were used according to the type of study. For in vitro studies, the SciRAP tool was used, which was adapted for its application in this type of approach. The JBI critical appraisal checklist was used to evaluate the risk of bias in clinical cases [7]. All evaluations were carried out by two evaluators independently; in the case of discrepancy, a third evaluator participated.

## 3. Results

**Figure 1:**
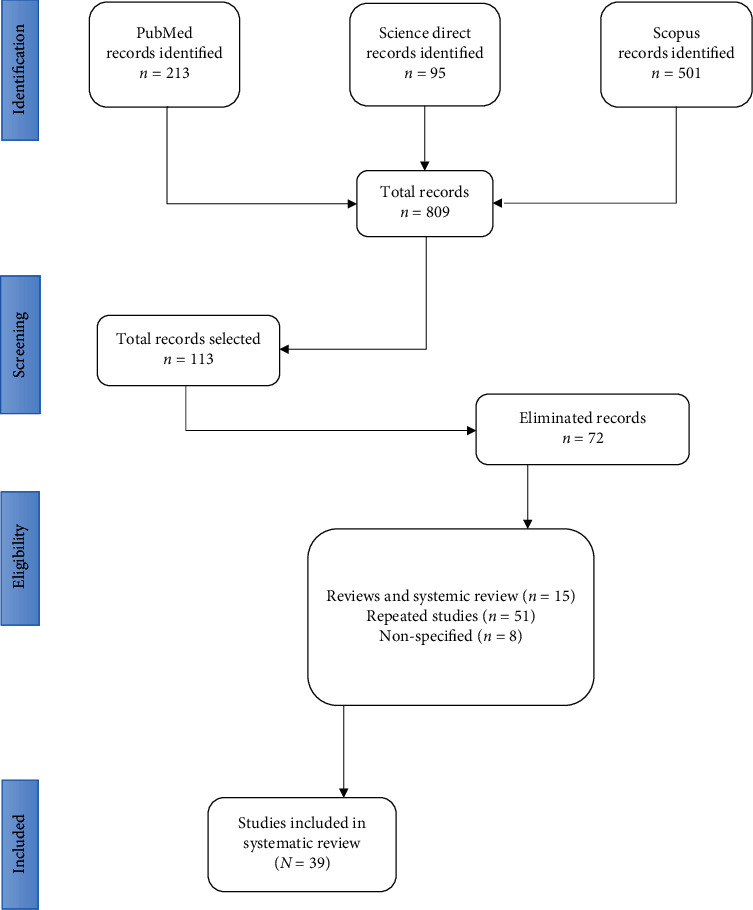
Search strategy used in the study, PRISMA flow chart.

The search strategy included characteristics of the studies, which initially analyzed the title and abstract; 696 studies were eliminated because they did not specify whether the research was on stem cells in COVID-19, leaving only 113 full-text studies. When these were analyzed, 72 were eliminated because they were review and systematic review studies, repeated studies, and others not specific to the topic of interest. After filtering according to the established search criteria, only 39 studies were included (PRISMA flow chart Figure 1).

### 3.1. Description of the Data

Of the total studies included (*n* = 39), 22 corresponded to in vitro investigations and 17 to human studies (clinical cases (*n* = 9), case series (*n* = 2), pilot clinical trials (*n* = 5), and clinical trials (*n* = 1)).

In vitro studies that induced pluripotent stem cells were the most used (*n* = 12). In clinical studies, stem cells derived from umbilical cord were the most reported (*n* = 11). The mean age of the study subjects was 58.3 years. After the application of stem cell therapy, the follow-up period was a minimum of 8 days and a maximum of 90 days (Supplementary) [8–38].

### 3.2. Risk of Bias

To determine the risk of bias, the SciRAP tool was used, which was adapted according to the studies to be evaluated; three fundamental aspects were evaluated: report quality, methodological quality, and study relevance. One hundred percent of the studies were identified as having low bias. The JBI critical appraisal checklist was used for case reports, which consists of eight questions focused on the description of the patient, their evaluation, the interventions carried out, and the adverse effects. All of the studies showed a low risk of bias (Table 1).

## 4. Discussion

One of the main characteristics of stem cells is their ability to modulate an innate and adaptive immune response due to the presence of proinflammatory receptors in their membrane, as well as their ability to synthesize IL-10, TGF-*β*, TSG-6, LIF, HGF, COX-2, prostaglandin-2, and others, in addition to promoting the regeneration and regulation of the expansion of immune cells [39]. This is why it has been a widely used model in the study of SARS-CoV-2.

The mechanism by which the virus enters the cell is through protein “S,” located on the surface of the membrane, by recognizing the ACE2 receptor located on the target cell [40]. Once recognition of the virus is carried out, transmembrane proteins (i.e., ADAM 17 or TMPRSS2) participate in the process of fusion and internalization of SARS-CoV-2 [41]. The evidence that exists relating to the expression of ACE2 and TMPRSS2 in stem cells indicates that stem cells from bone marrow and amniotic fluid have very little expression, and—in the case of adipose tissue stem cells—the expression decreases less than 10% without finding important changes in its expression when faced with inflammatory events [42], which indicates a low risk of infection of these cells to SARS-CoV-2. On the other hand, bioinformatics studies that compare the expression of ACE2 in stem cells of different origins have shown that the expression of ACE2 in adipose stem cells is higher compared to those of placental or umbilical cord origin. In addition, the low expression of ACE2 is related to a low expression of genes related to inflammasomes and immune regulation [43]. All of the above suggest that stem cells are a reliable therapeutic option due to the low expression of receptors for SARS-CoV-2 and that within the multiple sources of stem cells; those of fetal origin are the best therapeutic option. This is demonstrated in our review since the vast majority of clinical case studies focus on the effect of umbilical cord stem cells for the treatment of COVID-19 (*n* = 11).

In this study, pluripotent stem cells were identified as the cell type most used as a study model for SARS-CoV-2 (*n* = 12), specifically those that were induced. These derive from adult somatic cells that have been genetically reprogrammed into embryonic stem cells [44]. These cells have the capacity for self-renewal and differentiation to all cell types in the body; however, they have certain disadvantages depending on the technique used for their differentiation and the method based on the vector integration system. Despite being highly efficient, there is a risk that a cancer cell will develop [45]. By studying the susceptibility of different cell lines from induced stem cells, pancreatic and liver cells have been identified as being more susceptible to infection by SARS-CoV-2; on the other hand, cells that express ACE2—such as the endothelium, macrophages, and cortical neurons—show low or no permissiveness for the SARS-CoV-2 virus, suggesting that the nonlinear relationship between ACE2 and the permissiveness to SARS-CoV-2 infection highlights the importance of using pluripotent stem cells in place of cells that overexpress ACE2 to study the biology of SARS-CoV-2 [46].

As the lungs are the organ most affected by COVID-19, studies have been carried out that evaluate the response of pluripotent stem cells induced to an epithelial alveolar phenotype when infected with SARS-CoV-2, finding that they present a global transcriptomic change characterized by the expression of encoded cytokines by NF-*κ*B genes and epithelial interferon late responses [47]. Another study revealed that changes in RNA processing reducing the translation of 5′ capped mRNA and identified inhibitors of the mTOR and MAPK pathways, suggesting that SARS-CoV-2 induces arrest of the cell cycle, apoptosis, and damage to the nuclear envelope [48].

COVID-19 has affected millions of people around the world. The mildest symptoms range between fever and cough and/or digestive problems such as diarrhea and vomiting. In severe cases, there are pulmonary alterations with a respiratory rate of 30 per min and lower oxygen saturation below to 93% in addition to organ failure [49]; severe damage of this disease is characterized by an increase in the levels of inflammation mediators, such as cytokines and chemokines (IL-2, IL-7, IL-10; tumor necrosis factor) among others [ 50]. One of the markers of inflammation related to high mortality is IL-6; this suggests that high rates of mortality are related to the development of cytokine elevation syndrome induced by a cytokine storm [51]. In order to develop an immunological therapy that helps contain the side effects of SARS-CoV-2, clinical trials have been developed based on stem cell therapy.

It has been reported that the use of stem cells as immune therapy is related to the development of fever, without disclosing significant adverse effects [52]. When evaluating the safety and efficacy of stem cells from umbilical cord, it has been confirmed that patients do not develop side effects of consideration at a follow-up of 28 days. In addition, a decrease in the expression of IL-6 was observed [53], which is related to severe symptoms of COVID-19. On the other hand, when analyzing the clinical efficacy of the application of umbilical cord stem cells, it was observed that in patients with severe symptoms, the sensation of chest tightness, shortness of breath, and fatigue were improved. In the same way, the level of IL-6 and C-reactive proteins decreased from the third day of umbilical stem cell application, and a significant improvement from the seventh day of applying the therapy was reported [54]. Similarly, Guo et al. reported that when administering umbilical stem cells at a dose of 1 × 10^6^ cells per kilogram of weight, oxygenation levels are restored in patients with severe disease without reporting secondary reactions [55].

Umbilical stem cells have the characteristic that their isolation is safe and risk-free for the donor; their long-term cryopreservation does not affect their function or viability, and these types of cells carry a low possibility of transmission of viral infections. They present excellent immunological properties due to the fact that they exhibit HLA class I and II antigens related to the interferon-gamma response, which is a central regulator of the defense system that mediates the innate and adaptive immune response [56, 57].

Although positive effects have been reported on the use of stem cells as a therapeutic option for COVID-19, the reported absence of side effects after its application has been for relatively short periods of time, which is why it is necessary to carry out long-term follow-up to advance the processes of establishing stem cells as a therapeutic option.

The two main limitations of this study are as follows: (1) because of the methodology employed, the sample was not very large, and (2) the follow-up period of the clinical studies was relatively short. New studies with longer observation periods are emerging every day because of the importance of this current issue.

## 5. Conclusion

The SARS-CoV-2 infection develops intense inflammation due to the cytokine storm that develops. In addition to the sequelae that it generates in the tissues, prolonged inflammatory states can trigger the loss of partial function of the affected organ. The use of stem cells is a highly relevant therapeutic option because in vitro studies and clinical trials have shown that it counteracts the excessive secretion of cytokines.

## Figures and Tables

**(a):** 

In vitro studies			
Author	Reporting quality	Methodological quality	Relevance
Huang J.	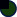	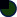	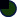
Jacob F.	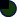	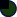	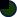
Bin Yu	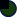	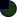	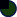
Wong CK	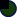	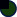	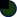
Yang L	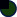	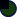	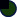
Youk J	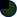	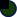	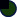
Ropa J	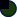	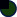	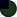
Cao Y	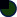	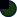	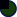
Sharma A	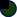	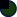	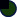
Surendran H	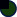	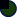	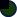
Schäfer R	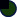	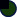	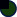
Katsura H	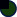	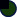	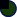
Purwati	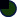	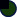	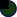
Kase Y.	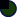	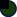	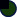
Ghazizadeh Z	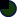	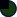	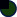
Hekman R	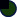	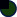	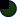
Kwon Y	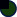	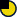	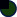
Duan F	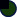	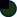	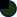
Han Y	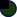	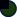	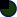

**(b):** 

Clinical studies									
Author	Q1	Q2	Q3	Q4	Q5	Q6	Q7	Q8	Quality
Tao J	y	y	y	y	y	y	y	y	High
Peng H	y	y	y	y	y	y	y	y	High
Liang B	Y	Y	Y	Y	Y	Y	Y	Y	High
Zengin R	y	y	y	y	y	y	y	y	High
Zhu Y	y	y	y	y	y	y	y	y	High
Zhang Y	y	y	y	y	y	y	y	y	High
Fisler G	y	y	y	y	y	y	y	y	High
Soler Rich R	y	y	y	y	y	y	y	y	High
Lázaro Del Campo P	y	y	y	y	y	y	y	y	High

## Data Availability

All data obtained from this study can be found in the Institute of Biomedical Sciences of the Autonomous University of Ciudad Juarez, Chihuahua, and can be requested through the corresponding author.

## Supplementary Materials

Supplementary Materials The information collected in the selected studies is shown in the following table; the information was divided according to the type of study, in vitro or clinical study. For in vitro studies, there is information about the main author, country, origin of stem cells, type of study, objective of the study, and conclusion. The clinical studies contain information related to the author, country, type of study, whether it is a clinical report, clinical series, or pilot study, origin of the stem cells, objective of the study, number of subjects included in the study, treatment plan, follow-up time to the established treatment plan, and the results obtained from the study.
