# Supplementary material for: Stem Cells as a Model of Study of SARS-CoV-2 and COVID-19: A Systematic Review of the Literature
Source: Biomed Res Int. 2021 Aug 25;2021:9915927. doi: 10.1155/2021/9915927 (PMC8390136; doi:10.1155/2021/9915927)
Supplement: Supplementary Materials — The information collected in the selected studies is shown in the following table; the information was divided according to the type of study, in vitro or clinical study. For in vitro studies, there is information about the main author, country, origin of stem cells, type of study, objective of the study, and conclusion. The clinical studies contain information related to the author, country, type of study, whether it is a clinical report, clinical series, or pilot study, origin of the stem cells, objective of the study, number of subjects included in the study, treatment plan, follow-up time to the established treatment plan, and the results obtained from the study. [file 9915927.f1.docx]

| **AUTOR**  *IN VITRO* STUDIES | **COUNTRY** | **STEM CELL ORIGEN** | **STUDY TYPE** | **MAIN OF THE STUDY** | **CONCLUSION** |
| --- | --- | --- | --- | --- | --- |
| Valyaeva et al., 2020 ^27^ | Rusia | Lung stem cells | *in silico* | To determine whether lung stem cells can be infected by SARS-CoV-2 by analzing published RNA-seq datasets | Epithelial stem cells express Ace2 and other SARS-CoV-2 entry factors, making these cells probable targets of SARS-CoV-2 infection. cell differentiation is accompanied by depletion of the ACE2 protein |
| [Huang](https://www.ncbi.nlm.nih.gov/pubmed/?term=Huang%20J%5BAuthor%5D&cauthor=true&cauthor_uid=32637964) et al., 2020 ^16^ | EUA | Pluripotent Stem Cell-Derived Human Lung Alveolar Type 2 Cells | *in vitro* | Report the successful infection of a pure population of human iPSC-derivedAT2-like cells with SARS-CoV-2, providing a reductionist model that reveals the cell-intrinsic distal lung epithelial global transcriptomic responses to infection | By 1 day post-infection(dpi), SARS-CoV-2 induced secretion of cytokines encodedby NF-kB target genes, by 4 dpi, there were time-dependentepithelial interferon responses and progressive loss of themature lung alveolar epithelial program |
| Jacob et al. 2020 ^28^ | EUA | Human Pluripotent Stem Cell-Derived Neural Cells | *in vitro* | To use hiPSC-derived neurons, astro-cytes, and microglia in monolayer cultures and region-specificbrain organoids of the cerebral cortex, hippocampus, hypothal-amus, and midbrain to investigate the susceptibility of brain cellsto SARS-CoV-2 infection | Observed sparse infection of neurons and astrocytes, with the exception of regions of organoids with choroid plexus epithelial cells that exhibited high levels ofinfectivity. Using an optimized protocol to generate choroidplexus organoids (CPOs) from hiPSCs, we showed evidence ofproductive infection by SARS-CoV-2 and functional conse-quences at cellular and molecular levels |
| Yu et al., 2020 ^26^ | EUA | Neural Stem Cell-Derived | *in vitro* | Evaluate the Innate and Adaptive Immunity of Murine Neural Stem Cell-Derived piRNA Exosomes/Microvesicles against Pseudotyped SARS-CoV-2 and HIV-Based Lentivirus | Murine NSCs produce exosomes/microvesicles (Ex/Mv) to provide innate and adaptive antiviral actions. Furthermore, they found that these NSC Ex/Mv are characterized by producing large libraries of P element-induced wimpy testis (PIWI)-interacting RNAs (piRNAs) against the genomes of various viruses, |
| Wong et al., 2020 ^29^ | China | Human-induced pluripotent stem cell-derived cardiomyocytes (hiPSC-CMs) | *in vitro* | hiPSC-CMs to study SARS-CoV-2 susceptibility and possible cytopathogenic effects. | hiPSC-CM platform can be used to recapitulate SARS-CoV-2-related myocardial injury, ranging from susceptibility, to direct cytopathogenic effects and inflammatory cytokine/chemokine response |
| Yang L. et al, 2020 ^15^ | EUA | Human pluripotent stem cells (hPSCs) | *in vitro* | Human pluripotent stem cells (hPSCs), including human embryonic stem cells (hESCs) and induced pluripotent stem cells (hiPSCs), can be used to derive functional human cells/tissues/organoids to systematically explore the viral tropism of SARS-CoV-2 and cellular responses to infection. | Generated a library of hPSC-derived cells/organoids, including pancreatic endocrine cells, liver organoids, endothelial cells, cardiomyocytes, macrophages, microglia, cortical neurons, and dopaminergic neurons, to evaluate the permissiveness of normal human cells to SARS-CoV-2 infection. The non-linear relationship between ACE2 and permissiveness to SARS-CoV-2 infection highlights the importance of using hPSC-derived primary-like cells instead of ACE2-overexpressing cells to study SARS-CoV-2 biology |
| Youk et al., 2020 ^31^ | Republic of Korea | Human Alveolar Stem Cell | *in vitro* | Develop a technique for long-term, feeder-free human three-dimensional (3D) alveolar type 2 cell cultures (hereafter referred to as h3ACs) established from single primary hAT2 cells that serve as stem cells in adult alveolar tissues | The study highlights the advantages of h3AC models to elucidate the pathogenesis of SARS-CoV-2 infection in alveolar stem cells |
| Ropa et al., 2021 ^32^ | EUA | Cord blood derived HSCs/HPCs and in peripheral blood derived immune cell subtype | *in vitro* | Examined the expression of ACE2, to which SARS-CoV-2 Spike (S) protein binds to facilitate viral entry | Confirm expression of ACE2 in subpopulations of HSCs and examine a full panel of primitive and lineage committed progenitor cells as well as fully mature immune cells for ACE2 expression. Also demonstrate marked suppressive effects on HPC functional colony forming cell capacity, reduced expansion of HSCs/HPCs, and physiological changes induced in peripheral blood (PB) cells when cells are grown in the presence of recombinant SARS-CoV-2 S protein. |
| Cao et al., 2020 ^33^ | China | Human mesenchymal stem cells | *in vivo* | Sought to examine the constitutive or inducible expression of ACE2 or TMPRSS2 during the MSC therapy process. | Results showed a ubiquitously low expression pattern of ACE2 among different donors, thus, it is necessary to select MSCs according to the low level of ACE2 and TMPRESS2 in the therapeutic practice to ascertain the safety by excluding the rare case of SARS-CoV-2 infection. |
| Sharma et al., 2020 ^34^ | EUA | Human induced pluripotent stem cell-derived cardiomyocytes (hiPSC-CMs | *in vitro* | Utilize human induced pluripotent stem cell-derived cardiomyocytes (hiPSC-CMs) as a model to examine the mechanisms of cardiomyocyte-specific infection by SARS-CoV-2. | Reduction in cardiac-specific contractile genes such as TNNT2 likely reflects the impairment in contractility of infected hiPSC-CMs, as well as overall CM death. |
| Surendran, Nandakumar & Pal, 2020 ^35^ | EUA | Human induced pluripotent stem cells | *in vitro* | To derive lung epithelial lineage from human induced pluripotent stem cells (hiPSC), to serve as a human model. | Show preliminary data on how human induced pluripotent stem cells-derived lung epithelial cell system could emerge as a relevant and sensitive platform for modeling SARS-CoV-2 infection and drug screening. |
| Schäfer et al., 2020 ^11^ | Germany | MSCs from bone marrow, amniotic fluid, and adipose | *in vitro* | Tested the potential of SARS-CoV-2 to infect human MSCs from different sources and to replicate within these cells | Observed very low expression of the current known entry molecules, ACE2 and TMPRSS2, on the MSC surface, as well as resistance of MSCs to SARS-CoV-2 infection. |
| Desterke et al., 2020 ^12^ | France | Adult bone marrow, adipose tissue or umbilical cord‐derived MSC | *in silico* | Bioinformatics and molecular analyses performed in several sources of MSCs from adult, fetal tissue and pluripotent stem cells evaluating the expression of ACE2 and the assessment of their signaling pathways associated with their anti‐inflammatory activity | ACE2 expression was found to be significantly higher in MSC‐derived from adipose tissue and adult bone marrow, MSCs expressing lower levels of ACE2 were also found to be associated with lower expression of genes involved in inflammasome and immune regulation. culture conditions can also impact ACE2 expression levels as after late passages (3‐5 passages). |
| Katsura et al., 2020 ^36^ | EUA | Human Lung Stem Cell | *in vitro* | Developed chemically defined conditions for human AT2 expansion and differentiation in alveolosphere cultures | SARS-CoV-2 infection of the defined AT2 alveolosphere system revealed an induction of autocrine and presumed paracrine IFN signaling and inflammatory pathways that blocked surfactant production and proliferation and induced cell death. |
| Purwati et al., 2020 ^37^ | Indonesia | Hematopoietic Stem Cells | *in vitro* | Conduct an in vitro study concerning the potency of hematopoietic stem cells (HSCs) and natural killer (NK) cells in handling COVID-19 | The findings findicate that HSCs can eliminate viruses in cells and supernatants after 24 hours of inoculation and NK cells play a critical role in decreasing the viral load of the SARS-CoV-2 virus |
| Kase et al., 2020 ^38^ | Japan | Human induced pluripotent stem cell | *in vitro* | To studie the mechanism that triggers the COVID-19-associated central nervous system disorders | Their findings suggest that the damage to the CNS caused by COVID-19 might be reduced by suppressing expression of CCN11 (cysteine-rich protein 61 (Cyr61)) |
| Ghazizadeh et al., 2020 ^39^ | EUA | Human embryonic stem cell derived cardiac cells. | *in vitro / in silico* | To identify candidate drugs capable of modulating ACE2 protein levels | Results provide key insights into ACE2 regulatory mechanisms, present strong molecular and clinical evidence for the role of androgen signaling in SARS-CoV-2 infection and identify potential therapeutic candidates for the treatment of COVID-19 |
| Hekman et al., 2020 ^17^ | EUA | Induced pluripotent stem cell-derived AT2s (iAT2s) | *in vitro / in silico* | Performed a deep quantitative temporal phospho/proteomic analysis to quantify cytopathologic changes induced by SARS-CoV-2 infection in iAT2s | Observed a DNA damage response in infected iAT2s, characterized by increased ATR kinase by 6 hpi, hypoactive cell cycle kinases, and direct phosphoproteomic evidence of activated proapoptotic kinase CAMK2D, suggesting that SARS-CoV-2 disrupts multiple signaling modules to cause AT2 growth arrest and apoptosis, potentially contributing to pulmonary necrosis. |
| Kwon et al., 2020 ^40^ | EUA | Human induced pluripotent stem cell-derived cardiomyocytes | *in vitro* | Hypothesized that SARS-CoV-2-infected cells such as airway epithelial cells secrete EVs carrying viral genetic material that may be taken up by cardiomyocytes and establish an indirect route of SARS-CoV-2 genetic material transmission | Results collectively demonstrated that lung epithelial cells expressing SARS-CoV-2 genes can secrete EVs containing viral RNA fragments that can be detected in cardiomyocytes suggesting an indirect route of viral RNA delivery into cardiac cells via EVs. |
| Duan, et al., 2020 ^41^ | EUA | Human pluripotent stem cells | *in vitro* | Used directed differentiation of human pluripotent stem cells (hPSCs) to establish a lung and macrophage co-culture system and model the host-pathogen interaction and immune response caused by SARS-CoV-2 infection | Inhibiting viral entry into target cells using an ACE2 blocking antibody enhanced the activity of M2 macrophages, resulting in nearly complete clearance of virus and protection of lung cells |
| Li Y, Li H & Zhou, 2020 ^42^ | China | Human embryonic stem cell | *in silico* | To study the epigenetic regulation of ACE2 expression | Found that EZH2-mediated H3K27me3 inhibits ACE2 expression, and this pattern may be conserved among mammalian cells. |
| Han et al. 2021 ^43^ | EUA | Human pluripotent stem cells | *in vitro* | Developed a lung organoid model using human pluripotent stem cells (hPSC-LOs). | Found that multiple colonic cell types, especially enterocytes, express ACE2 and are permissive to SARS-CoV-2 infection and identified entry inhibitors of SARS-CoV-2, including imatinib, mycophenolic acid and quinacrine dihydrochloride. |

| **AUTOR**  CLINICAL STUDIES | **COUNTRY** | **TYPE STUDY** | **STEM CELL ORIGEN** | **MAIN OF THE STUDY** | **NO. CASES** | **MEAN AGE (YEARS)** | **TREATMENT PLAN** | **FOLLOW-UP TIME AFTER TREATMENT ADMINISTRATION** | **RESULTS** |
| --- | --- | --- | --- | --- | --- | --- | --- | --- | --- |
| Tao et al. 2020 ^44^ | China | Case report | Umbilical cord blood-derived mesenchymal stem cells | Report the umbilical cord blood-derived mesenchymal stem cells therapy for critically ill cases with COVID-19 | N/A | 72 | 1.5×106 USB-MSCs per kilogram of the patient’s weight were infused intravenously every 48 hours, with a total of five-time infusion | 10 Days | hematological and biochemical indexes, including lymphocytes and renal function improved. Pulmonary static compliance increased significantly and PaO2/FiO2 ratio maintained stable. |
| Peng et al., 2020 ^45^ | China | Case report | Umbilical cord mesenchymal stem cells | To know whether there is a coordinated relationship between convalescent plasma donors and MSCs in COVID-19 therapy. | N/A | 66 | Total number of infused cells was 1 × 106 cells per kilogram, once every 3 days, and 3 times. | 42 days | Intravenous infusion of Convalescent plasma and MSCs for the treatment of severe COVID-19 patients may have synergistic characteristics in inhibiting cytokine storm, promoting the repair of lung injury, and recovering pulmonary function. |
| Liang et al., 2020 ^46^ | China | Case report | Umbilical cord mesenchymal stem cells | To investigate the capabilities of hUCMSCs to modulate the immune response and repair the injured tissue. | N/A | 65 | The allogenic hUCMSCs were administrated intravenously 3 times (5 × 107 cells each time) on days 13, 16, and 19 | 30 days | The possible effects of hUCMSCs might be anti-inflammation and tissue repair to COVID-19 patients, and it was also reported that MSCs could down regulate proinflammatory cytokines and chemokines and increase IL-10 and VEGF which could promote the lung repair. |
| Tang et al., 2020 ^47^ | China | Clinical pilot study | Menstrual blood-derived MSCs | Uses menstrual blood-derived MSCs for the treatment of two patients with severe COVID-19 in Wuhan | 2 | 72.5 | Injection dose of MSCs was set to 1 million per kg body weight. The patient received MSC transplantation through intravenous infusion three times | 15 days | Found that the oxygenation indicators improved, the immune indicators increased, and a number of inflammation indicators decreased after MSC treatment. Chest computed tomography (CT) showed the adsorption of bilateral lung exudate lesions after MSC transplantation |
| Meng et al., 2020 ^22^ | Germany | Clinical trial | Human umbilical cord-derived mesenchymal stem cell | Performed a parallel assigned, controlled, non-randomized, phase 1 clinical trial to evaluate the safety of human umbilical cord-derived mesenchymal stem cells infusions in the treatment of patients with moderate and severe COVID-19 during the early phase of the COVID-19 pandemic | 18 | 47.3 | Three cycles of intravenous infusion of allogeneic UC-MSCs (3 × 107 cells each infusion) on days 0, 3, and 6. The total volume of the UC-MSCs infusion was 60 ml. | 28 days | The patients in the UC-MSCs-treatment group showed a decrease of serum IL-6, a gradual decline of IL-6 level might turn out to be a biologically relevant surrogate marker of the efficacy of MSCs treatment in patients with COVID-19. Patients with severe disease with the highest IL-6 level showed the biggest drop in the IL-6 level and improvement of oxygenation index, suggesting that UC-MSCs treatment might have the most benefit for individuals with high levels of inflammatory cytokines |
| Zengin et al., 2020 ^48^ | Turkey | Case report | Umbilical cord-derived MSCs | Report the case of a critically ill COVID-19 patient received an experimental treatment with MSC therapy in conjunction with recommended treatment protocols | N/A | 72 | 2 dosis via intratracheal and intravenous routes 0.7×106 cells/kg intravenous, 0.3×106 cells/kg intratracheal with 4 unites of heparin | 60 days | Following the MSC transplantation, the need for inotropic agents started to disappear, acidosis, electrolyte imbalance and hypoxemia started to improve, lung chest X-ray showed slight regression in the ground-glass imaged infiltration in the middle right lung periphery, and significant regression in the low-density infiltrations in the lower right lung and lateral left lung |
| Zhu et al., 2020 ^49^ | China | Case report | Umbilical Cord Mesenchymal Stem Cells | Introduce a COVID-19 case of a critically ill male patient in China. Then we explore the safety and effectiveness of UC-MSC treatment and provide a new potential means for COVID-19 | N/A | 48 | intravenous injection 1 × 106 cells per kilogram of weight. | 14 days | The absolute number of lymphocytes of the patient was significantly increased. they speculated that UC-MSCs might adjust the body’s immune function, to improve inflammatory reaction, improve lung function and multiple organ functions to improve the outcome of this critically ill patien |
| Zhang et al., 2020 ^50^ | China | Case report | Umbilical cord Wharton’s jelly-derived MSCs | report a case of a severe COVID-19 patient treated with human umbilical cord Wharton’s jelly-derived MSCs (hWJCs) | N/A | 54 | intravenous infusion 1 × 106 cells per kilogram of weight | 8 days | The serum C-reactive protein and inflammatory factors (IL-6 and TNF-α) of the patient were gradually reduced |
| Shu et al., 2020 ^23^ | China | Clinical pilot study | Umbilical cord mesenchymal stem cell | To evaluate the efficacy of hUC-MSCs for treating severe COVID-19 | 12 | 65 | Intravenous administration 2 × 106 cells/kg | 15 days | MSC therapy can suppress excessive immune system activation and promote endogenous repair by improving the microenvironment |
| Feng, et al., 2020 ^51^ | China | Clinical pilot study | Umbilical cord MSCs | To evaluate the feasibility and safety of intravenous infusion of umbilical cord MSCs (UC‐MSCs) in severe and critically severe COVID‐19 patients | 19 | 61.7 | Four rounds of transplantation in total, with one‐day intervals in between 1 × 108 cells once | 28 days | The improvement of radiological presentations, recovery of lymphocyte count and decrease of cytokine levels |
| Leng et al., 2020 ^52^ | China | Clinical pilot study | clinical grade MSC | MSC transplantation pilot study to explore their therapeutic potential for HCoV-19 infected patients | 7 | 57 | intravenous injection 1 × 106 cells per kilogram of weight | 14 days | The transplantation of MSCs improved the outcome of COVID-2019 patients may be due to regulating inflammatory response and promoting tissue repair and regeneration |
| Sánchez-Guijo et al., 2020 ^53^ | Spain | Case series | Adipose tissue derived mesenchymal stromal cells | To determine whether the administration of adiposetissue derived mesenchymal stromal cells (AT-MSC) is safe and potentially useful in these patients | 13 | 60 | 2 or 3 intravenous administration dosis of 1 x 106 AT-MSCs/kg of recipient’s body weight. | 16 days | MSC derived from adipose tissue can be safely administered in critically ill patients with COVID-19 pneumonia and that administration of AT-MSC was followed by clinical improvement and changes in inflammatory and immune populations, which suggest a potential biological effect of the cells. |
| Fisler et al., 2020 ^54^ | EUA | Case report | haplo-identical hematopoietic stem cell (HSTC) | Present an adolescent patient who contracted severe acute respiratory syndrome coronavirus 2 one week after a paternal haplo-identical hematopoietic stem cell transplant | N/A | 15 |  |  | Present a teenager post-HSCT with COVID-19-induced hyperferritinemic acute lung injury, multisystem organ dysfunction, and MALS (macrophage activation-like syndrome) |
| Soler Rich, Rius Tarruella & Melgosa Camarero, 2020 ^55^ | Spain | Case report | células mesenquimales de médula ósea alogénica | Mostrar el abordaje terapéutico del SARS-CoV-2 (COVID-19) mediante células mesenquimales de médula ósea alogénica expandidas. | N/A |  | Vía intravenosa una dosis de 80 x 10 E6MSC de médula ósea alogénica (1 x 10 E6MSC/kg de peso) | 14 days | Aún asumiendo el rol que pudo jugar la inmunidad natural, estando discutidos los efectos beneficiosos de la hidroxicloroquina, especulamos que el efecto inmunomodulador y proregenerativo de la administración intravenosa de altas dosis de MSC expandidas pudiera haber sido el principal responsable del favorable curso clínico, biológico y radiológico del caso. |
| Malhotra et al., 2020 ^56^ | Australia | Clinical pilot study | Umbilical cord blood cells | To undertake a pilot, feasibility RCT of umbilical cord blood derived cell therapy for treatment of adult patients infected with SARS-CoV-2 virus related moderate-to-severe pneumonia. | 24 |  | Intravenous injection of expanded umbilical cord blood cells at a dose of 5 million cells/kg (maximum dose - 500 million cells). | 90 days | Safety and tolerability of cell administration within first 24 hours of administration; clinical improvement on a seven-category clinical improvement ordinal scale. |
| Guo et al., 2020 ^24^ | China | Case series | umbilical cord mesenchymal stem cells | to report their experience using UC-MSCs for the treatment of severe COVID-19 pneumonia | 31 | 70 | Intravenous drip of UC-MSCs (1 × 106 cells per kilogram of weight) | 10.7 days | UC-MSC therapy may restore oxygenation and downregulate cytokine storms in patients hospitalized with severe COVID-19 without any infusion reaction |
| Lázaro Del Campo et al., 2020 ^57^ | Spain | Case report | Hematopoietic Cell Transplantation | To report blood products and specifically bone marrow can cause transfusion-transmitted infection | N/A | 57 |  |  | Hematopoietic Cell Transplantation from a donor with positive SARS-CoV-2 nasopharyngeal PCR in the asymptomatic incubation period does not cause COVID-19 in the recipient |

The information collected in the selected studies is shown in the following table, the information was divided according to the type of study, in vitro or clinical study. for in vitro studies, there is information about the main author, country, origin of stem cells, type of study, objective of the study and conclusion. The clinical studies contain information related to the author, country, type of study, whether it is a clinical report, clinical series or pilot study, origin of the stem cells, objective of the study, number of subjects included in the study, treatment plan, follow-up time to the established treatment plan and the results obtained from the study.
